# Ghrelin Aggravates Prostate Enlargement in Rats with Testosterone-Induced Benign Prostatic Hyperplasia, Stromal Cell Proliferation, and Smooth Muscle Contraction in Human Prostate Tissues

**DOI:** 10.1155/2019/4748312

**Published:** 2019-11-22

**Authors:** Xiaolong Wang, Yiming Wang, Christian Gratzke, Christian Sterr, Qingfeng Yu, Bingsheng Li, Frank Strittmatter, Annika Herlemann, Alexander Tamalunas, Beata Rutz, Anna Ciotkowska, Raphaela Waidelich, Chunxiao Liu, Christian G. Stief, Martin Hennenberg

**Affiliations:** ^1^Department of Urology, Ludwig-Maximilians University, Munich, Germany; ^2^Department of Urology, Zhujiang Hospital, Southern Medical University, Guangzhou, China; ^3^Department of Urology, University of Freiburg, Freiburg, Germany

## Abstract

Epidemiologic studies revealed a context between lower urinary tract symptoms (LUTS) suggestive of benign prostatic hyperplasia (BPH) and metabolic syndrome. However, molecular mechanisms underlying this relationship are largely unknown. Prostate enlargement and increased prostate smooth muscle tone are important factors in the pathophysiology of LUTS suggestive of BPH. In the present study, we studied effects of the metabolic hormone ghrelin on prostate enlargement in rats with experimentally induced BPH, growth of cultured stromal cells from human prostate (WPMY-1), and smooth muscle contraction of human prostate tissues. Ghrelin (20 nmol/kg daily, p.o., 2 weeks) increased prostate size in rats with testosterone-induced BPH. Microarray identified 114 ghrelin-upregulated genes (2-fold or more) in these prostates, with possible roles in growth, smooth muscle contraction, or metabolism. 12 genes were selected for further analyses. In human prostate tissues, mRNA levels of 11 of them correlated positively with ghrelin receptor (GHSR) expression, but only two with the degree of BPH. Accordingly, no correlation was evident between GHSR expression level and BPH in human prostate tissues. In WPMY-1 cells, the GHRS agonist MK0677 upregulated 11 of the selected genes. MK0677 induced proliferation of WPMY-1 cells, shown by EdU assay, colony formation, proliferation markers, flow cytometry, and viability. In myographic measurements, GHSR agonists enhanced contractions of human prostate strips. Together, ghrelin may aggravate prostate enlargement, stromal cell growth, and prostate smooth muscle contraction in BPH. Ghrelin may deteriorate urethral obstruction independently from BPH, qualifying the ghrelin system as an attractive new target to be tested for LUTS treatment in BPH.

## 1. Introduction

Benign prostatic hyperplasia (BPH) is commonly associated with lower urinary tract symptoms (LUTS), resulting from urethral obstruction due to prostate enlargement and increased prostate smooth muscle tone [1]. Treatment becomes often inevitable and is aimed (1) at improving symptoms and (2) at preventing BPH progression and complications [2]. Widespread options for medical treatment of LUTS suggestive of BPH are *α*_1_-adrenoceptor antagonists and phosphodiesterase-5 inhibitors, which may improve symptoms by inhibition of prostate smooth muscle contraction, and 5*α*-reductase inhibitors to reduce prostate growth [2]. Combination therapies are commonly applied to address prostate size and symptoms at once [2]. However, available medical therapies have limited efficacy even in mild to moderate LUTS [2]. Improvements of symptoms and of urinary flow by *α*_1_-blockers are restricted to 50% [1, 3, 4]. Similarly, the risk of symptomatic progression can be reduced at most by 35-40% by monotherapy and by 66% by combination therapies [3].

Epidemiological studies suggested a context between metabolic syndrome and BPH/LUTS [5]. Metabolic syndrome itself and its single components were identified as risk factors for BPH and LUTS [5]. While this association is well established at a clinical level, molecular links of this relationship are poorly understood. Preclinical studies focused on the role of inflammatory mediators and lipoproteins [5]. Any role of metabolic hormones, which contribute to the metabolic syndrome as well, has not been considered to explain molecular connections between metabolic syndrome and BPH [5].

Adipokines, including ghrelin or other metabolic hormones play a central role for pathophysiology of the metabolic syndrome. Ghrelin is a metabolic peptide hormone acting by activation of the growth hormone secretagogue receptor 1a (GHSR1a) and probably via additional receptors [6]. Ghrelin increases food intake and body weight [6, 7]. At a cellular level, ghrelin induces proliferation, differentiation, and lipid accumulation in adipocytes and preadipocytes [6]. Regulation and promotion of cell cycle in other cell types including smooth muscle cells outside the lower urinary tract have been reported [6, 8, 9]. Most ghrelin functions are attributed to GHSR activation by acyl-ghrelin, while desacyl-ghrelin does not activate the GHSR [6]. In obese subjects with metabolic syndrome, the ratio between acyl- and desacyl-ghrelin is shifted towards acetylated ghrelin, underlining the role of ghrelin for the metabolic syndrome [10]. Considering the relationships between metabolic syndrome and BPH on the one hand and the role of smooth muscle contraction and prostate growth for LUTS suggestive of BPH on the other hand, a possible role of the ghrelin system for BPH and LUTS may be assumed. Here, we report effects of ghrelin and GHSR agonists on prostate growth and gene expression in rats with experimentally induced BPH and on proliferation, gene expression, and smooth muscle contraction in human prostate tissues and in cultured stromal cells.

## 2. Materials and Methods

### 2.1. Testosterone-Induced BPH in Rats

Procedures in rats were approved by the Animal Experimentation Ethics Committee of the Southern Medical University (SMU), Guangzhou, China. Procedures applied in our study were in accordance with the regulations applying for countries of the European Union. Animal experiments took place in the Laboratory Animal Center, Southern Medical University, Guangzhou, China. Six-week-old male Sprague Dawley rats (180-220 g) were purchased from the Laboratory Animal Center of SMU and housed under controlled conditions of temperature, humidity, and light/dark cycle. Water and food were supplied ad libitum. After 72 h of accommodation, rats were distributed randomly into three groups (*n* = 8/group): (1) a sham-operated group (sham) undergoing sham operation for castration, subcutaneous injection of corn oil, and peroral treatment with vehicle (ghrelin control, distilled water); (2) an androgen-induced BPH group (BPH) undergoing castration, subcutaneous injection of dihydrotestosterone (3 mg/kg) for four weeks, and peroral treatment with vehicle (distilled water); and (3) an orally ghrelin-treated group (BPH+ghrelin) undergoing castration, subcutaneous injection of dihydrotestosterone (3 mg/kg) for four weeks, and peroral treatment with rat ghrelin (20 nmol/kg) (Tocris, Bristol, UK) for two weeks, starting two weeks after initiation of testosterone treatment. Injections and oral treatments were performed once daily, starting after 72 h of the surgical procedure. A complete randomization process was carried out so that every experimental unit had an equal chance of receiving each treatment. Ghrelin was dissolved in distilled water and applied by gavage. In previous studies, application of 12 or 16 nmol/kg of ghrelin induced growth hormone release and functional effects in vivo in rats [11–13]. After 4 weeks, rats were sacrificed by cervical dislocation, following anesthesia using carbon dioxide prior to cervical dislocation, and prostates were removed, shock frozen, and stored at -80°C until microarray analyses. Body weight was measured before the experiments and after the 4-week treatment and wet weight of the isolated prostate directly following prostatectomy.

### 2.2. Microarray Analysis

A global gene expression analysis of rat prostates was performed according to the Agilent One-Color Microarray-Based Gene Expression Analysis protocol (Agilent Technologies, Santa Clara, CA, USA). Total RNA was linearly amplified and labeled with Cy3-UTP. Concentration and specific activity of the labeled cRNAs (pmol Cy3/*μ*g cRNA) were measured by NanoDrop ND-1000. Labeled cRNA was fragmented and diluted with GE hybridization buffer. Hybridized arrays were scanned with the Agilent DNA Microarray Scanner (part number G2505C). Quantile normalization and subsequent data processing were performed with using the GeneSpring GX v12.1 software package (Agilent Technologies).

All data obtained by microarray in this study have been deposited in NCBI's Gene Expression Omnibus and are accessible through the GEO repository with the accession number GSE129561 (https://www.ncbi.nlm.nih.gov/geo/query/acc.cgi?acc=GSE129561). The data sets could be downloaded from the GEO database.

Selection of genes to categories of interest was performed by definition of predetermined categories. These categories were defined before the experiments. Criteria for selection for subsequent validation in cell culture and human prostate tissues (including correlation analyses) were a 1-fold or higher upregulation together with an assumed function belonging to at least one of the three categories below. Thus, selection of genes for subsequent validation was based on predefined categories and on the results from microarrays but was not done before the experiments. Predefined categories included “smooth muscle contraction,” “growth,” and “metabolism.” Categorization to “smooth muscle contraction” was based on the assumption that smooth muscle contraction depends on a panel of functions including actin and extracellular matrix organization, adhesion (of the cytoskeleton to membranes and of cells to extracellular matrix), neurotransmitter release, and myosin light chain phosphorylation and on a panel of signaling pathways including G protein-coupled receptors, heterotrimeric G proteins, small monomeric GTPases, RhoA/Rho kinase, calcium, nitric oxide, and others [1]. Categorization to “growth” was based on a function in proliferation, survival, regulation of cell cycle, or differentiation. Searches for information of gene functions and gene ontology was performed using different data bases, including https://omim.org, https://genecards.org, and https://geneontology.org.

### 2.3. Human Prostate Tissues

Human prostate tissues were obtained from patients who underwent radical prostatectomy for prostate cancer (*n* = 44) as previously described [1]. This study was carried out in accordance with the Declaration of Helsinki of the World Medical Association and has been approved by the ethics committee of the Ludwig-Maximilians University, Munich, Germany. Informed consent was obtained from all patients. All samples and data were collected and analyzed anonymously. Patients with previous ablative surgery of the prostate were excluded. Tissues were taken solely from the periurethral zone, considering that most prostate cancers arise in the peripheral zone [14, 15]. Prostates showing tumors in the periurethral zone upon macroscopic inspection (found in less than 1% of prostates undergoing this sampling procedure) were not subjected to sampling. BPH is present in ca. 80% of patients with prostate cancer [16, 17].

### 2.4. Real-Time Polymerase Chain Reaction (RT-PCR)

RNA isolation from frozen prostate tissues or from cells and RT-PCR were performed as previously described using primers provided by Qiagen (Hilden, Germany) [18]. Results were expressed using the *ΔΔ*CP method, where number of cycles (Ct) at which the fluorescence signal exceeded a defined threshold for GAPDH was subtracted from Ct values for target mRNAs (Ct_target_‐Ct_GAPDH_ = ΔCP), and values were calculated as 2^-*Δ*CP^. Correlation analyses were performed using the 2^-*Δ*CP^ values from concerning genes obtained from the same samples. In stimulation experiments performed in WPMY-1 cells, values of stimulated samples were expressed as fold of the mean of control samples.

### 2.5. Tension Measurements

Smooth muscle contraction of human prostate strips was performed as previously described in tissue baths with four chambers (Danish Myotechnology, Aarhus, Denmark) [18]. Dimethylsulfoxid (DMSO) for controls, L-692,585, or MK0677 was added 30 min before contractions and was induced by electric field stimulation (EFS) or by *α*_1_-adrenoceptor agonists. In each experiment, tissue from the same prostate was allocated to all four channels, and DMSO or one of the GHSR agonists was added to two channels following determination of KCl-induced contractions and subsequent washout. Only one curve was recorded with each sample. For calculation of agonist-induced contractions, tensions were expressed as % of KCl-induced contractions, which were induced by addition of a 2 M KCl solution to obtain a final high molar KCl concentration of 80 mM. All samples (19 tissues, 4 samples per tissue) contracted in response to high molar KCl, resulting in maximum KCl-induced contractions of 52.6 ± 46.8 mN (mean ± standard deviation). *E*_max_ and EC_50_ values were calculated by curve fitting for each single experiment using GraphPad Prism 6 (Statcon, Witzenhausen, Germany) and analyzed as described below.

### 2.6. Culture and Stimulation of WPMY-1 Cells

WPMY-1 cells were obtained from American Type Culture Collection (ATCC; Manassas, VA, USA) and cultured, stimulated, and processed as previously described [18].

### 2.7. Plate Colony Assay

About 100 cells were placed to each well of a 6-well culture plate and treated with MK0677 in indicated concentrations. Cells were incubated at 37°C for 14 days, washed twice with phosphate-buffered saline (PBS), and fixed by 2 ml of 10% trichloroacetic acid (TCA) overnight (4°C). Subsequently, all plates were washed five times with cold water and stained with 0.4% sulforhodamine B (SRB) solution (diluted in 1% acetic acid) at room temperature for 30 minutes. Before taking photos for counting and documentation of representative wells, all plates were labelled and washed five times with 1% acetic acid. The number of all colonies (with a size of 1 mm ore more) was counted manually with assistance from ImageJ from pictures taken from whole wells. Colonies growing close to each other and merging with each other were counted as one colony.

### 2.8. Flow Cytometry

For detection of cell cycle phase distribution by flow cytometry, WPMY-1 cells (10^5^ cells/well) were grown overnight in 6-well plates. Subsequently, the medium was replaced by a serum-free medium, and cells were preincubated with and without MK-0677 (100 nM). After 24 h or 48 h, preincubation media were removed; cells were rinsed with PBS for harvesting, fixed with ethanol, and centrifuged (500 g, 5 min). Pellets were suspended in 1 ml of precooled PBS for 1 min, centrifuged (500 g, 5 min), and suspended in 200 *μ*l RNase staining solution containing propidium iodide (PI) (Abcam, Cambridge, UK), which was added to each individual tube. Fluorescence was measured by flow cytometry using a FACSCalibur (Beckton Dicksinson, Franklin Lake, NJ, USA). Suitable forward scatter (FSC) vs. side scatter (SSC) was established to exclude debris and cell aggregates, and PI fluorescence signals in the FL2 channel using 488 nm laser illumination were collected. For each tube, the third-party Mod Fit LTTM software (Verity Software House, Torpsham, ME, USA) was used for data acquisition (1 × 10^4^ events for each sample) and for analysis. Markers were set up on a histogram plot to delineate <2N, 2N (G1 stage), 2N-4N (DNA synthesis), 4N (mitotic), and >4N intensity regions. Results were expressed as % of cells in different phases.

### 2.9. EdU Assay

5-Ethynyl-2′-deoxyuridine (EdU) assay in MK0677- or solvent-stimulated WPMY-1 cells was performed as previously described using the “EdU-Click 555” cell proliferation assay (Baseclick, Tutzing, Germany) [18]. Results were expressed as % of proliferating cells from the whole examined population.

### 2.10. CCK-8 Assay

Viability of MK0677 in WPMY-1 cells was assessed as previously described using the cell counting kit 8 (CCK-8) (Sigma-Aldrich, St. Louis, MO, USA) [18]. Results were expressed as means of the optical density from all independent experiments.

### 2.11. Data and Statistical Analysis

Data are presented as means ± standard deviation (SD) with the indicated number (*n*) of independent experiments. Wherever this was feasible, all single values from all independent experiments or all samples are shown together with means. Statistical analyses were performed by nonparametric tests, with the exception for group comparisons in frequency and concentration response curves, where a Gaussian distribution may be assumed (2-way ANOVA). For comparison of paired groups from datasets containing two groups, the Wilcoxon Rank Sum test was applied. For comparison of datasets containing three related, correlating groups, the Friedman test was used. If the test was significant, a nonparametric post hoc test (Stepwise Stepdown Multiple Comparisons) was used for multiple comparisons, i.e., to locate which pairs are different. For comparison of datasets containing three unrelated, unpaired (i.e., independent) groups, 1-way ANOVA was applied. If the test was significant, Fisher's Least Significant Difference (LSD) test was used for multiple comparisons, i.e., to compare two groups within the dataset of three groups. All tests were performed using SPSS® version 20 (IBM SPSS Statistics, IBM Corporation, Armonk, New York, USA). *p* values < 0.05 were considered statistically significant. The present study and analyses were designed to be exploratory, but not designed to test a prespecified statistical null hypothesis. Therefore, *p* values reported here should be considered as descriptive and not as hypothesis testing. All groups included in the statistical analyses were based on five or more independent experiments, so that minimum group size subjected to statistical tests was *n* = 5, and all groups being compared with each other by statistical tests showed identical group sizes. In fact, despite the explorative design, the preplanned group sizes were *n* = 5 for cell culture experiments, and *n* = 5 or higher for other experiments, if the application of statistical analysis for descriptive *p* values was intended. Microarray analyses were based on group sizes of *n* = 3, so that no statistical tests were applied to these data. Series of organ bath experiments were finished as soon as meaningful results (with a descriptive *p* value < 0.05) were obtained with group sizes of *n* = 5 or more, according to previous recommendations [19, 20]. Considering validation of microarray-identified genes in human prostate tissues, the sample size of 12 was chosen for technical reasons, together with the availability of prostate tissues. No data or experiments were excluded from analyses. Spearman's correlation analysis was performed using GraphPad Prism 6 (Statcon, Witzenhausen, Germany). No correlation analyses were performed but not reported, e.g., because correlations lacked statistical significance.

### 2.12. Materials, Drugs, and Nomenclature

MK0677 (2-amino-N-[(1R)-2-[1,2-dihydro-1-(methylsulfonyl)spiro[3H-indole-3,4′-piperidin]-1′-yl]-2-oxo-1-[(phenylmethoxy)methyl]ethyl]-2-methylpropanamide methanesulfonate) and L-692,585 (3-[[(2R)-2-hydroxypropyl]amino]-3-methyl-N-[(3R)-2,3,4,5-tetrahydro-2-oxo-1-[[2′-(1H-tetrazol-5-yl)[1,1′-biphenyl]-4-yl]methyl]-1H-1-benzazepin-3-yl]-butanamide) are nonpeptide ghrelin receptor agonists. Stock solutions were prepared in DMSO and stored at -20°C until used. Aqueous stock solutions of noradrenaline and the *α*_1_-adrenoceptor agonist phenylephrine were freshly prepared before each experiment. MK0677 and L-692,858 were obtained from Tocris (Bristol, UK), and noradrenaline and phenylephrine were obtained from Sigma-Aldrich (Munich, Germany).

## 3. Results

### 3.1. Effects of Ghrelin on Prostate Size

**Figure 1:**
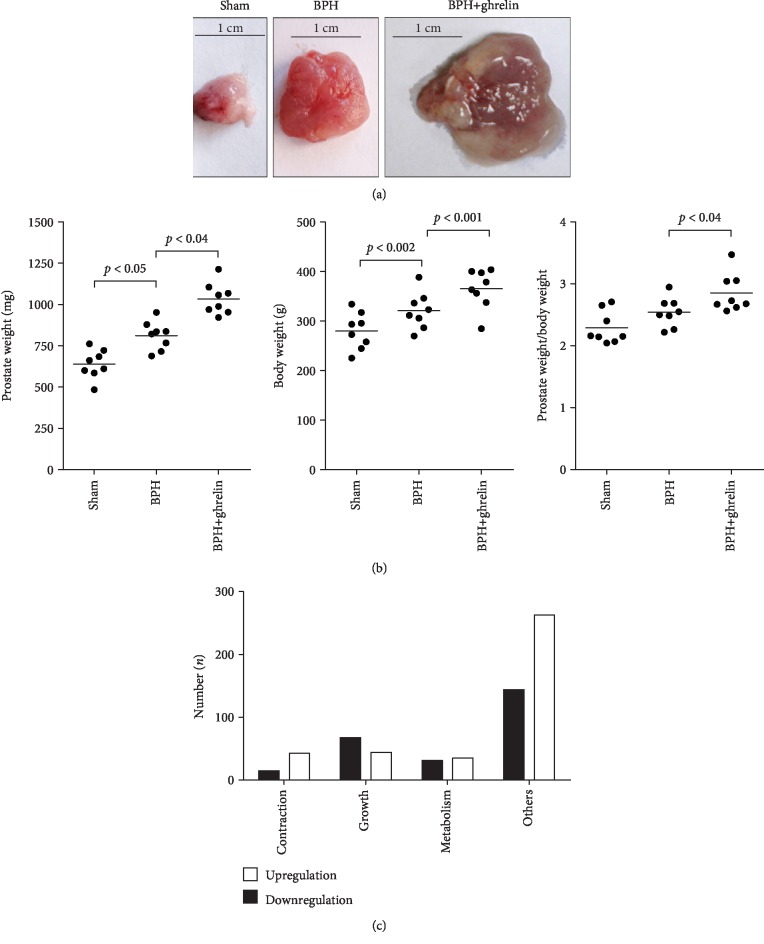
Ghrelin-induced regulation of prostate size and gene expression in rats with testosterone-induced BPH. (a) Testosterone supplementation (3 mg/kg b.w., s.c., daily) for four weeks induces prostate enlargement in rats (compare sham with BPH), which was aggravated by ghrelin (10 *μ*g/kg body weight, i.p., 14 d) (representative images, *n* = 8 rats/group). (b) Prostate weight, body weight, and the ratio of prostate and body weight in sham-treated, BPH, and ghrelin-treated BPH rats. Shown are data of all rats from a series including *n* = 8 rats/group and descriptive *p* values (Friedman two-way ANOVA by ranks). (c) Numbers and functions of ghrelin-regulated genes in prostates from rats with testosterone-induced BPH, identified by microarray analysis (*n* = 3 rats per group, i.e., BPH rats treated with ghrelin or vehicle). Putative function of genes, which were up- or downregulated 1-fold or more by ghrelin, was checked by research in databases. Subsequently, genes were sorted to indicated categories.

BPH and prostate enlargement in rats can be induced by castration and subsequent testosterone supplementation (four weeks), which was aggravated by application of ghrelin (Figure 1(a)). Two weeks after oral ghrelin or vehicle treatment, body weights in ghrelin-treated BPH rats were higher compared to the vehicle group (365 ± 40 g vs. 320 ± 37 g). This was paralleled by increased prostate wet weight (1.03 ± 0.096 g vs. 0.81 ± 0.086 g) and higher prostate body weight ratio in the ghrelin group compared to the vehicle group (2.85 ± 0.31 × 10^−3^ vs. 2.54 ± 0.24 × 10^−3^) (Figure 1(b)).

### 3.2. Effects of Ghrelin on Gene Expression in Rat Prostates

By microarray, we analyzed the effects of ghrelin on gene expression in prostates of rats with testosterone-induced BPH. The expression of 893 mRNAs was altered by ghrelin. 258 mRNAs represent genes, which were upregulated 1-fold or more by ghrelin (i.e., showing values 2-fold of controls). From these genes being upregulated at least 1-fold by ghrelin, 68 are genes for which a regulation of growth may be assumed, 15 are genes for which a role in regulation of smooth muscle contraction may be assumed, and 31 are genes for which a role in metabolism may be assumed (Figure 1(c)). 385 of the altered mRNAs represent genes, which were downregulated to 2-fold or less by ghrelin. From these genes being downregulated 1-fold or more by ghrelin, 44 are genes for which a regulation of growth may be assumed, 43 are genes for which a role in the regulation of smooth muscle contraction may be assumed, and 35 are genes for which a role in metabolism may be assumed (Figure 1(c)). Remaining mRNAs were altered less than 2-fold, or a primary function in growth, proliferation, or metabolism was not assumed from research in databases (Figure 1(c)).

### 3.3. Correlation of Ghrelin-Regulated Genes with BPH and GHSR Expression in Human Prostates

**Figure 2:**
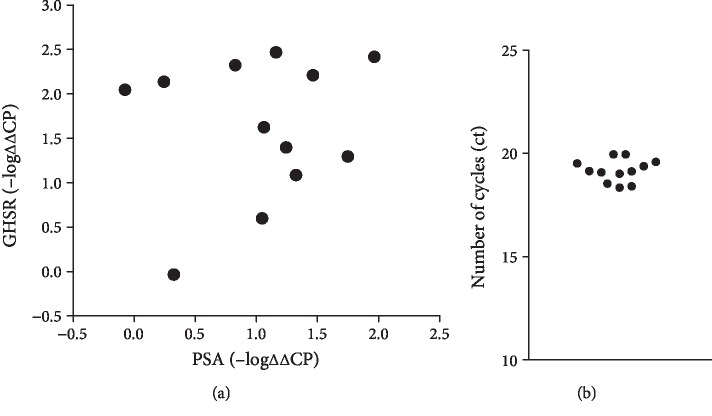
GHSR detection in human prostate tissue. (a) Correlation analysis for GHSR mRNA with PSA mRNA (-log*ΔΔ*CP), following RT-PCR of human prostate tissues. (b) Ct values for GAPDH, single values from all prostates included to this analysis.

Findings from microarray analyses of rat prostates were verified in human prostate tissues obtained from radical prostatectomy. In these tissues, we analyzed mRNA of twelve selected genes from all three categories, which were identified by microarray (Table 1) together with mRNA for GHSR and prostate-specific antigen (PSA), which increases with degree of BPH [18, 21]. Selected genes of the category “smooth muscle contraction” may be associated with regulation of RhoA or monomeric GTPases in general (*ARHGEF4*, *ABRA*), contraction-mediating kinases (*RTKN2*, *LIMK2*), cytoskeleton organization (*ABRA*), neurotransmission (*GAL*), or adhesion and extracellular matrix (*SPON1*). Selected genes of the category “growth” may be involved in proliferation (*TTK*, *CCNE1*, and *FOXM1*) or may be a growth factor (*BMP6*). Double functions and categorization to “growth” in addition to “smooth muscle contraction” appear feasible for *SPON1*, which may be involved in the regulation of proliferation besides extracellular matrix formation. Identified genes of the category “metabolism” (*SLC10A6*, *THEM5*) may be involved in fat metabolism, sugar transport, or lipid regulation. For 11 of the 12 selected genes, mRNA levels correlated positively with GHSR mRNA in human prostate tissues (*R* > 0.5) (Table 2). Positive correlation with PSA was only suggested for two of these genes (Table 2). Consistently, no correlation was evident between GHSR and PSA mRNAs following detection by RT-PCR, neither for *ΔΔ*CP values nor for –log*ΔΔ*CP values (*R* = 0.164 for *ΔΔ*CP, *R* = 0.151 for -log *ΔΔ*CP) (Figure 2(a)). Plotting of –log*ΔΔ*CP suggested two groups of samples, i.e., one with stable and high GHSR mRNA levels despite varying content of PSA mRNA and another with GHSR mRNA levels increasing with PSA mRNA (Figure 2(a)). The validity of variation of these values was confirmed by the low variation of Ct values for GAPDH, which was used as reference (Figure 2(b)).

### 3.4. MK0677-Induced Gene Expression in WPMY-1 Cells

**Figure 3:**
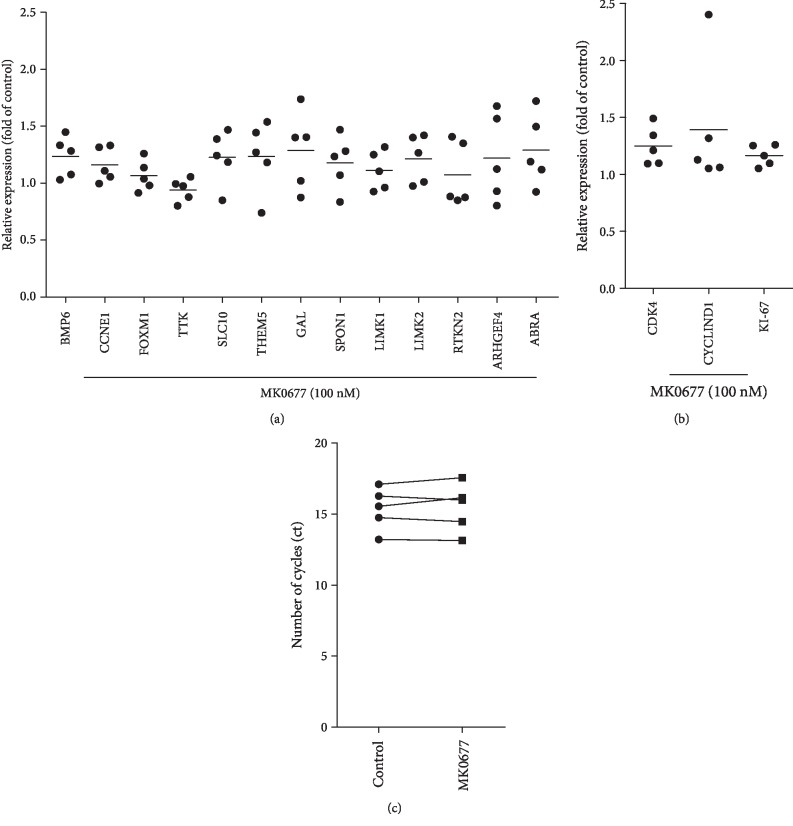
Effects of MK0677 on mRNA expression of selected genes in WPMY-1 cells. (a) Effects of MK0677 (24 h, 100 nM) on mRNA levels of genes selected from microarray in WPMY-1 cells, assessed by RT-PCR. Shown are relative expression levels compared to unstimulated controls (normalized to mean of controls, which is 1) (single values of stimulated samples, from each of five independent experiments, and mean for each gene as bar). (b) Effects of MK0677 (24 h, 100 nM) on mRNA levels of proliferation markers in WPMY-1 cells, assessed by RT-PCR. Shown are relative expression levels compared to unstimulated controls (normalized to mean of controls, which is 1) (single values of stimulated samples, from each of five independent experiments, and mean for each gene as bar). (c) Ct values for GAPDH in cells with and without (control) stimulation with MK0677 (24 h, 100 nM) (single values of samples from each of five independent experiments, with paired samples from each independent experiment connected by a line).

Ghrelin-dependent regulation of selected genes identified by microarray analyses of rat prostates (Table 1) and showing correlation with GHSR expression in human prostates was examined by RT-PCR in WPMY-1 cells. WPMY-1 is an immortalized cell line from human prostate stroma, which may express GHSR, and shows a smooth muscle cell-like phenotype including the expression of the smooth muscle marker calponin, but lacking cytokeratin expression [22, 23]. Stimulation with the GHSR agonist MK0677 induced upregulation (resulting in mRNA levels 1.1-fold or more of unstimulated controls) of 10 from 12 examined genes, including two of the category “growth” (*BMP6*, *CCNE1*), two of the category “metabolism” (*SLC10*, *THEM5*), and at least four of the category “smooth muscle contraction” (*GAL*, *SPON1*, *LIMK2*, and *ABRA*) (Figure 3(a)). Similar results were obtained for *LIMK1*, which was not selected based on microarray, but recently suggested to promote human prostate smooth muscle contraction (Figure 3(a)) [20]. Ct values for GAPDH, which was used for reference (housekeeping), were not changed by stimulation with MK0677 (average Ct 15.37 ± 1.488 in controls, 15.45 ± 1.699 in MK0677-stimulated cells) (Figure 3(c)).

### 3.5. MK0677-Induced Proliferation of WPMY-1 Cells

**Figure 4:**
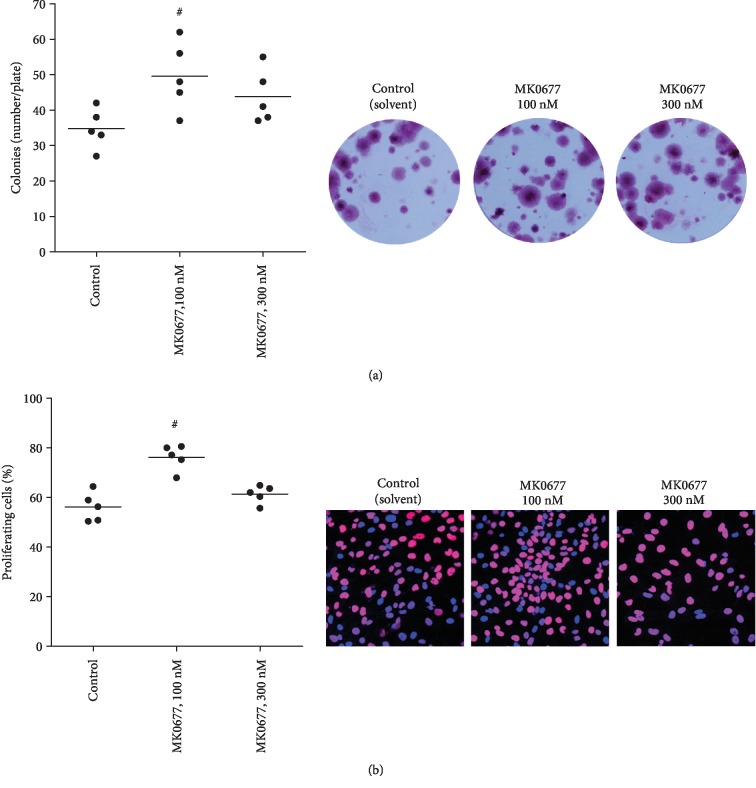
Effects of MK0677 on proliferation in WPMY-1 cells. (a) Effects of MK0677 (14 d) in plate colony assay (single values from each of five independent experiments, with mean for each gene as bar, and representative plates) (^#^*p* < 0.04 vs. control after Friedman two-way ANOVA by ranks). (b) Effects of MK0677 (24 h) on proliferation, assessed by EdU assay (single values from each of five independent experiments, with mean for each gene as bar, and representative images) (^#^*p* < 0.02 vs. control after Friedman two-way ANOVA by ranks).

**Figure 5:**
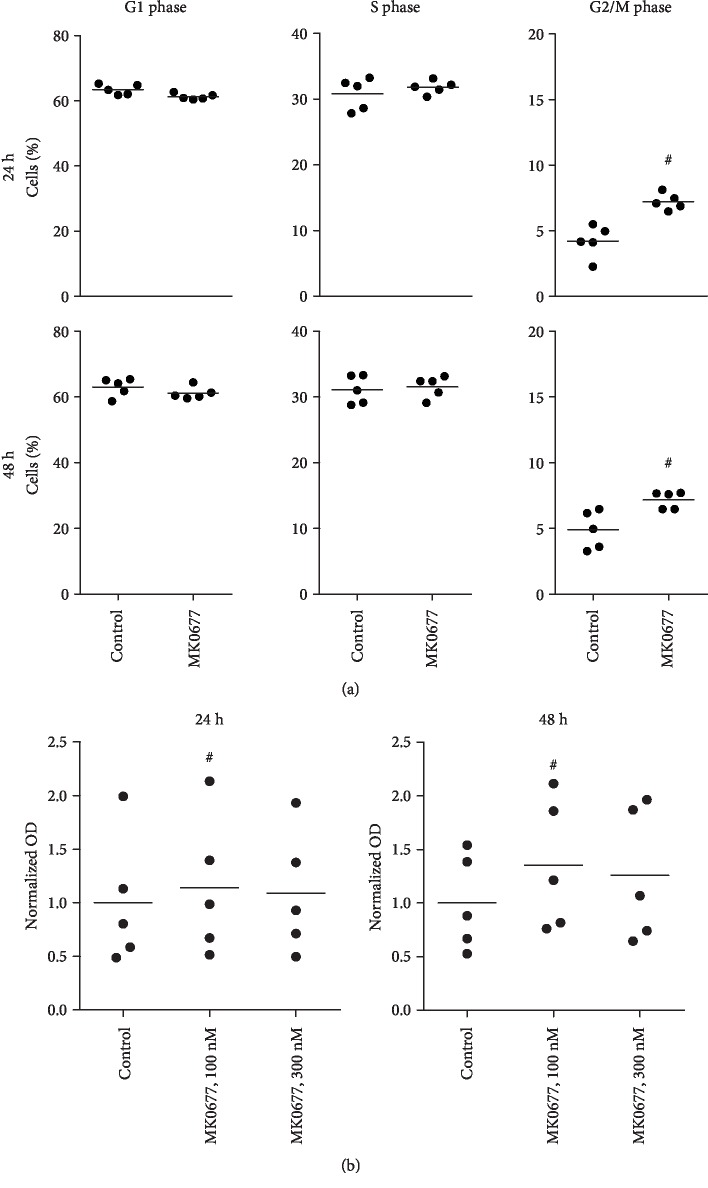
Effects of MK0677 on cell cycle and viability in WPMY-1 cells. (a) Effects of MK0677 (100 nM, 24-48 h) on cell cycle phases, assessed by flow cytometry analyses. Shown are representative experiments (left panels), and single values from each of five independent experiments, with mean for each gene as bar (^#^*p* < 0.05 vs. control after Wilcoxon Rank Sum test). In figures for representative experiments, the G1 phase is represented by the first red peak, the G2/M phase by the second red peak, and the S phase by the hatched areas. (b) Effects of MK0677 on viability, assessed by CCK-8 assays (single values from each of five independent experiments, with mean for each gene as bar) (*p* < 0.04 vs. control after Friedman two-way ANOVA by ranks).

Proliferation of WPMY-1 cells was assessed using several readouts (Figures 2 and 3). Stimulation with MK0677 (100 nM, 24 h) increased the mRNA levels of three proliferation markers (Cdk4, cyclinD1, and Ki-67) (Figure 3(b)). Proliferation was confirmed by plate colony assay (Figure 4(a)) and EdU assay (Figure 4(b)), where MK0677 induced proliferation. Promotion of cell cycle was again demonstrated by flow cytometry, which suggested increased fractions of cells in the G2/M phase and decreased fractions of cells in the G1 phase following stimulation with MK0677 (Figure 5(a)). In line with the MK0677-induced proliferation, CCK-8 assays demonstrated that MK0677 does not induce cytotoxic effects but rather increases the viability of WPMY-1 cells (Figure 5(b)).

### 3.6. Effects of GHSR Agonists on Human Prostate Smooth Muscle Contraction

**Figure 6:**
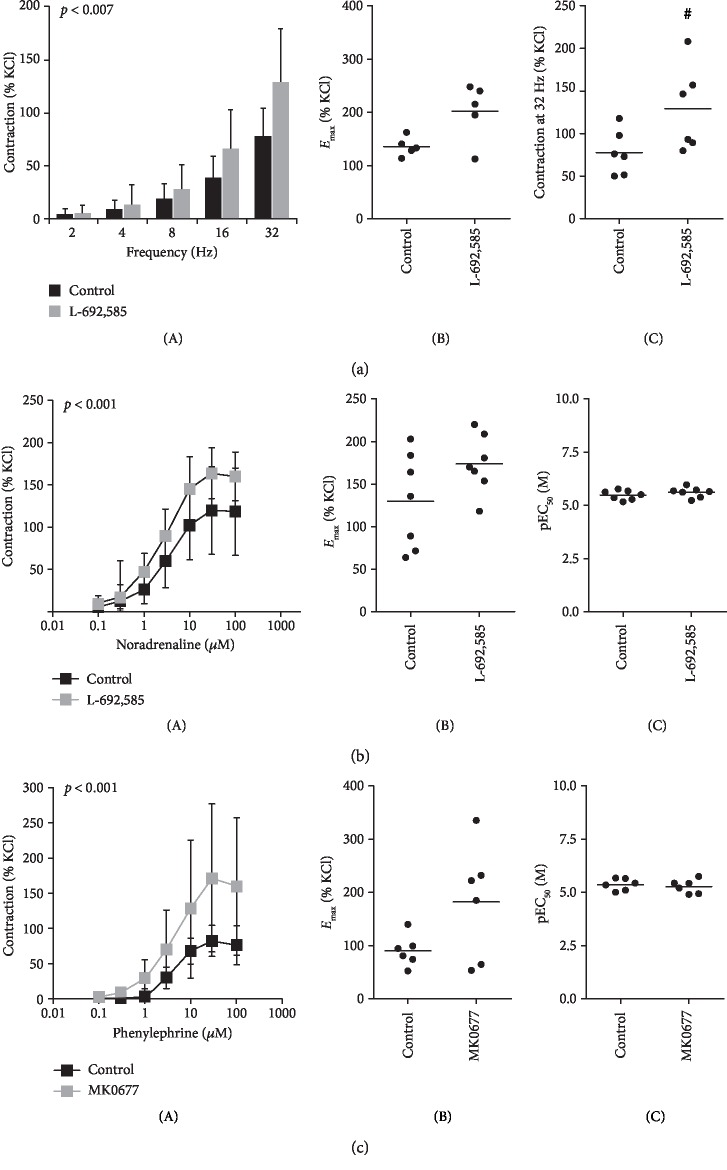
Effects of GHSR agonists on contractility of human prostate tissues. (a) Effects of L-692,585 (300 nM) on contractions of human prostate strips induced by electric field stimulation (tissues from *n* = 6 patients). (b) Effects of L-692,585 (300 nM) on contractions of human prostate strips induced by noradrenaline (tissues from *n* = 7 patients). (c) Effects of MK0677 (100 nM) on contractions of human prostate strips induced by the *α*_1_-adrenoceptor agonist phenylephrine (tissues from *n* = 6 patients). In all series, tissue from each patient being allocated to control and GHSR agonist group in each experiment. Data are mean ± SD in frequency- and concentration-response curves (*p* value from 2-way ANOVA for comparison between groups as indicated in frequency and concentration response curves) (A), *E*_max_ values for each single experiment (calculated by curve fitting) in (b) and (c) (B), and from 5 of 6 experiments in (a, B) (as feasible calculation was not possible for one experiment) (^#^*p* < 0.03 vs. control after Wilcoxon Rank Sum test), single values for 32 hertz-induced contractions in (a, C), and pEC_50_ values for each single experiment (calculated by curve fitting) in (b) and (c) (C).

Effects of GHSR agonists on contractions of human prostate strips were assessed by myographic measurements. L-692,585 enhanced contractions induced by electric field stimulation, which are mediated by adrenergic neurotransmission (Figure 6(a)). This was confirmed by changed settings, where L-692,585 and MK0677 enhanced noradrenaline- and phenylephrine-induced contractions (Figures 6(b) and 6(c)). *E*_max_ values, which were calculated by curve fitting for each separate experiment and group (Figure 6), confirm the increases of EFS-, noradrenaline-, and phenylephrine-induced contractions by L-692,585 and MK0677 seen in concentration- and frequency-response curves. L-692,585 and MK0677 were without effects of EC_50_ values of noradrenaline and phenylephrine (Figure 6).

## 4. Discussion

Our findings demonstrate that GHSR activation promotes prostate growth and prostate smooth muscle contraction in BPH, being imparted by genomic and nongenomic GHSR effects. Consequently, ghrelin may aggravate prostate enlargement, prostate smooth muscle tone, and finally urethral obstruction in patients with BPH. This may take place independent from the degree of BPH, so that ghrelin may deteriorate hyperplastic growth and bladder outlet obstruction in addition to BPH, e.g., in patients with BPH and metabolic syndrome. This may represent a mechanism explaining the association of metabolic syndrome with BPH and/or LUTS, which was previously evidenced by epidemiologic studies.

A main finding of our study is that treatment with ghrelin increased prostate size in rats with experimentally induced BPH. In BPH, prostate enlargement and increased prostate smooth muscle tone may both contribute to urethral compression and symptoms due to impaired bladder emptying [1]. By microarray, we identified genes, which were upregulated by ghrelin in prostates of rats with BPH and for which a role in smooth muscle contraction and proliferation may be assumed. We validated these findings (1) in cultured stromal cells, where expression of most of these genes was upregulated by GHSR activation, and (2) in human prostate tissues, where expression levels of most of these genes correlated with GHSR expression. Interestingly, a correlation with PSA expression or a correlation of GHSR expression with PSA content was not observed in human prostate tissues, leading to the conclusion that ghrelin actions may occur independently from the degree of BPH in the (hyperplastic) prostate. We assume that ghrelin may not necessarily cause BPH itself. Rather, our findings suggest that it may aggravate BPH and the factors underlying LUTS suggestive of BPH. Thus, in patients with BPH, increased GHSR activity and/or metabolic syndrome may increase the risk for progression of BPH. Finally, our data may point to two groups with different relationships between GHSR expression and BPH, although an overall correlation was not observed. Thus, an allocation to groups with stable, high GHSR expression despite varying PSA on the one hand and with a GHSR expression increasing with PSA on the other hand appears possible. Confirming and characterization of these relationships should be a subject of future studies and require higher sample sizes as in our explorative study.

Our findings suggest that GHSR activation may promote prostate smooth muscle contraction by genomic actions and nongenomic actions. Thus, ghrelin-induced upregulation of procontractile genes was paralleled by increases of smooth muscle contraction in the organ bath following short-time exposure of human prostate strips to GHSR agonists. It appears possible that both can take place in vivo, where increased prostate smooth muscle tone is a major factor contributing to urethral obstruction and LUTS suggestive of BPH [24, 25]. We observed similar results using different GHSR agonists (MK0677, L-692,585) and different *α*_1_-adrenoceptor agonists (phenylephrine, noradrenaline), so that these different settings may confirm each other. In the prostate, adrenergic neurotransmission causes smooth muscle contraction by activation of postsynaptic *α*_1_-adrenoceptors. Accordingly, neurogenic contractions induced by EFS were enhanced as well by L-692,585. It has previously been shown that MK0677 and L-692,585 may both activate phospholipase C followed by inositol-1,4,5-trisphosphate production and may induce calcium mobilization [26–29]. Both processes mediate smooth muscle contraction in the prostate [1]. Consequently, it appears possible that MK0677 and L-692,585 promoted *α*_1_-adrenergic contractions of prostate strips by these pathways.

In WPMY-1 cells, MK0677-induced proliferation was confirmed by different readouts. Ghrelin-mediated proliferation has been well-documented for different cell types [6, 7]. Regarding smooth muscle cells outside the prostate, effects of ghrelin on proliferation and cell cycle have been reported for vascular smooth muscle cells, although with conflicting results. Several studies pointed to an inhibition of proliferation by ghrelin in vascular smooth muscle cells, which may oppose our findings in WPMY-1 cells [30–32]. Other findings may suggest the opposite, as ghrelin inhibited apoptosis of vascular smooth muscle cells, and mice with GHSR deficiency showed reduced numbers of smooth muscle cells in the neointima of arteries with wire-induced injury [31, 33]. Together, our data suggest that ghrelin promotes growth of prostate cells, which is supported by cell culture experiments and in vivo using a rat model with experimentally induced BPH.

Our findings in isolated tissues and cell culture were obtained using small molecule GHSR agonists. MK0677 and L-692,585 were developed as orally available, potent growth hormone secretagogues. Both compounds resulted from optimization of preceding lead compounds to induce growth hormone release in rat pituitary cell assays, where MK0677 showed an EC_50_ of 1.3 nM and L-692,585 of 3 nM [34, 35]. Oral availability of MK0677 and L-692,585 was first confirmed in dog models [36–38]. Following the discovery of the GHSR, both compounds were identified as agonists of this receptor. MK0677 activates GHSR-coupled pathways and induces GHSR-mediated effects with EC_50_ values in the nanomolar range (0.2-1.4 nM) [27]. In contrast to their efficacies in the nanomolar range, receptor binding may differ between MK0677 and L-692,585. MK0677 was identified as a competitive receptor agonist, while L-692,585 may act by mixed mechanisms including a strong allosteric component of action [27, 28]. Thus, Ki values of 6.5 nM for MK0677, but of 2.5 *μ*M for L-692,585, were reported from ghrelin competition assay [27, 28]. GHSR binding of MK0677 and activation of receptor-coupled signaling pathways have been intensively studied. With regard to receptor binding, affinity, potency, and signaling activation, MK0677 strongly resembles endogenous acyl-ghrelin, probably much more than other nonpeptide GHSR agonists do [27, 39, 40]. Obviously, GHSR1a is currently the only known target for MK0677. In fact, MK0677 does not bind the splice variant GHSR1b [41]. On the other hand, binding to other G protein-coupled receptors has to the best of our knowledge not been examined. However, as MK0677 activates the GHSR with a very similar behaviour than ghrelin peptides, most of its effects may be mediated by this receptor.

Considering our findings suggesting aggravation of prostate growth, stromal cell proliferation, and smooth muscle contraction by ghrelin, it appears possible that patients with BPH/LUTS may profit from ghrelin management. The tolerability of several small molecule GHSR ligands has been confirmed in clinical studies [6]. Most promising may be inverse agonists, as the efficacy of pure antagonists is limited by high intrinsic GHSR activity [6, 42, 43]. It may be anticipated that GHSR antagonism (or inverse agonism, respectively) reduces prostate enlargement and smooth muscle tone simultaneously, so that clinical studies with inverse agonists may be attractive in patients with LUTS suggestive of BPH. Cardiovascular side effects of *α*_1_-blockers resulting from vasorelaxation often limit medical therapy and may account for discontinuation rates up to 65% 12 months after first prescription [2, 44]. Ghrelin peptides or GHSR agonists do not show hemodynamic effects or induce hypotension, so that adverse events similar to those of *α*_1_-blockers may not be expected from inverse GHSR antagonists [45–47]. Currently, combination therapies with 5*α*-reductase inhibitors and *α*_1_-blockers are required to address prostate enlargement and symptoms at once [2]. From GHSR antagonists or inverse agonists, both may be expected at once. Novel medications are of high demand, considering the insufficient efficacy of current options, high discontinuation rates, and the foreseeable rise in patients' numbers. The ghrelin system and other metabolic hormones merit further consideration in the context of LUTS suggestive of BPH.

## Figures and Tables

**Table 1:** Ghrelin-upregulated genes in hyperplastic rat prostates, selected from microarray for further analysis in human prostate tissues and in WPMY-1 cells, grouped to functional categories. Rats with testosterone-induced BPH were treated with ghrelin (20 nmol/kg, daily) or vehicle. Prostates were harvested four weeks after initiation of ghrelin treatment and subjected to microarray analysis (prostates from *n* = 3 animals per group). The selection represents genes, which were upregulated 1-fold or more by ghrelin treatment (fold change) and for which a function for growth, smooth muscle contraction, or metabolism appears likely.

Gene	Fold change	Simplified description
Growth		
BMP6	8.764	TGF-beta receptor agonist
TTK	4.258	Proliferation-promoting kinase
CCNE1	4.187	Cyclin E1
FOXM1	2.649	Transcriptional activator involved in proliferation
Contraction		
GAL	10.13	Regulation of neurotransmission
SPON1	3.111	ECM, adhesion, vascular smooth muscle
LIMK2	2.432	Smooth muscle contraction in hyperplastic human prostate
RTKN2	2.418	Rhotekin 2, effector of contraction-mediating Rho kinase
ARHGEF4	2.267	Rho guanine nucleotide exchange factor
ABRA	2.049	Actin-binding Rho activating protein
Metabolism		
SLC10A6	5.006	Metabolism, sugar import into cell
THEM5	4.300	Metabolism, lipid regulation

**Table 2:** Correlation analysis for mRNAs of genes selected from microarray analysis with mRNAs of GHSR and PSA, based on mRNA detection by RT-PCR in human prostate tissues (samples from 12 patients for each gene).

Gene	Correlation coefficient (Spearman's *r*)	
	With GHSR	With PSA
BMP6	0.965	-0.124
TTK	0.999	0.913
CCNE1	0.959	0.063
FOXM1	0.987	-0.016
GAL	0.903	-0.389
SPON1	0.324	-0.044
LIMK1	0.536	0.514
RTKN2	0.826	-0.369
ARHGEF4	0.679	0.452
ABRA	0.995	-0.252
SLC10A6	0.997	-0.25
THEM5	0.992	-0.209

## Data Availability

All data used to support the findings of this study are included within the article.

## Abbreviations

BPH: Benign prostatic hyperplasia
LUTS: Lower urinary tract symptoms
GHSR: Growth hormone secretagogue receptor
TURP: Transurethral resection of the prostate
PSA: Prostate-specific antigen
EFS: Electric field stimulation
FACS: Fluorescence-activated cell sorting
FSC: Forward scatter
SSC: Side scatter
PI: Propidium iodide
EdU: 5-Ethynyl-2′-deoxyuridine
CCK-8: Cell counting kit 8
SRB: Sulforhodamine B
GTP: Guanosine-5′-triphosphate
GEO: Gene expression omnibus.
